# RFCS-YOLO: Target Detection Algorithm in Adverse Weather Conditions via Receptive Field Enhancement and Cross-Scale Fusion

**DOI:** 10.3390/s25030912

**Published:** 2025-02-03

**Authors:** Gang Liu, Yingzheng Huang, Shuguang Yan, Enxiang Hou

**Affiliations:** 1 Jiangsu Province Engineering Research Center of Photonic Devices and System Integration for Communication Sensing Convergence, Wuxi University, Wuxi 214105, China; 2 School of Electronics and Information Engineering, Nanjing University of Information Science & Technology, Nanjing 210044, China; 202212490587@nuist.edu.cn (Y.H.); 202212490551@nuist.edu.cn (S.Y.); 202212490581@nuist.edu.cn (E.H.)

**Keywords:** receptive field enhancement, cross-scale fusion, attention mechanism, YOLOv7, loss function

## Abstract

The paper proposes a model based on receptive field enhancement and cross-scale fusion (RFCS-YOLO). It addresses challenges like complex backgrounds and problems of missing and mis-detecting traffic targets in bad weather. First, an efficient feature extraction module (EFEM) is created. It reconfigures the backbone network. This helps to make the receptive field better and improves its ability to extract features of targets at different scales. Next, a cross-scale fusion module (CSF) is introduced. It uses the receptive field coordinate attention mechanism (RFCA) to fuse information from different scales well. It also filters out noise and background information that might interfere. Also, a new Focaler-Minimum Point Distance Intersection over Union (F-MPDIoU) loss function is proposed. It makes the model converge faster and deals with issues of leakage and false detection. Experiments were conducted on the expanded Vehicle Detection in Adverse Weather Nature dataset (DWAN). The results show significant improvements compared to the conventional You Only Look Once v7 (YOLOv7) model. The mean Average Precision (mAP@0.5), precision, and recall are enhanced by 4.2%, 8.3%, and 1.4%, respectively. The mean Average Precision is 86.5%. The frame rate is 68 frames per second (FPS), which meets the requirements for real-time detection. A generalization experiment was conducted using the autonomous driving dataset SODA10M. The mAP@0.5 achieved 56.7%, which is a 3.6% improvement over the original model. This result demonstrates the good generalization ability of the proposed method.

## 1. Introduction

With the continuous progress of artificial intelligence, automatic driving technology has garnered a lot of attention in recent years. People are worried about vehicle safety when traveling [1]. So, traffic target detection has become more important. It is a key part of automatic driving technology. Thanks to deep learning [2], traffic target detection in normal weather has made great progress. But when there are bad weather conditions like rain, snow, haze, wind, or sandstorms, the performance of target detection algorithms is badly affected. Low visibility and environmental interference are big challenges for the vehicle detection system [3]. So, detecting traffic targets in bad weather is very important for road traffic safety and the development of automatic driving technology [4].

Recently, deep learning has developed fast and pushed forward research on target detection [5]. Now, deep learning-based target detection algorithms can be mainly divided into two types: two-stage target detection algorithms and single-stage target detection algorithms. Two-stage algorithms, such as the Faster Region-based Convolutional Neural Network (Faster R-CNN) [6], can detect objects more accurately. However, they tend to be slow. These algorithms first find the areas that might have detection targets through the network model. Then, they classify and adjust these areas to achieve the detection results. On the other hand, single-stage algorithms, such as Single Shot Detection (SSD) [7] and the YOLO series [8,9,10], can detect things faster, but their accuracy is lower. These methods work in an end-to-end way. That means a single network takes the input image data and gives out the output objects, including where the objects are and what type they are. Although two-stage algorithms can detect things accurately, they are too slow for real-time use. However, single-stage algorithms are popular in target detection because they have fewer parameters and can detect things faster. People are still trying to improve their detection accuracy and performance. For example, Wang et al. [11] proposed YOLOv9 and introduced the Generalized Efficient Layer Aggregation Network (GELAN) architecture, enabling the model to capture multi-scale information more effectively. Meanwhile, they put forward Programmable Gradient Information (PGI) to solve the problem of the loss of gradient information during the backpropagation process. Wang et al. [12] proposed YOLOv10 and put forward a dual assignment strategy. This strategy circumvents the Non-Maximum Suppression (NMS) algorithm, avoids generating redundant detection boxes, and reduces the inference delay.

Many scholars have put forward different vehicle and pedestrian detection algorithms for different situations. Under normal weather conditions, Wei et al. [13] proposed a lightweight decoupled prediction head. It classifies and regresses feature layers of different sizes. It improves the detection ability of small targets in road scenes. Yan et al. [14] designed a brand-new Focused Diffusion Feature Pyramid Structure based on the YOLOV8 algorithm. This enables the model to focus more on feature information and reduce feature redundancy. Li et al. [15] introduced the Repc3 (Reparameterization Convolutional Block 3) module into the YOLOv8 algorithm to strengthen the capabilities of feature integration and information enhancement. Meanwhile, they adopted the aggregation-distribution mechanism to achieve multi-scale feature fusion and enhance the ability to perceive changes in multi-scale images. Yuan et al. [16] improved the DETR (Detection Transformer) algorithm. They selectively extracted high-quality feature information and enhanced the detection ability for different occluding parts of the detection targets.

They have obtained good results in accuracy and speed. But in bad weather conditions, problems like blurry images and poor visibility stop target detection. To solve these problems, Liu et al. [17] designed a fully adaptable image processing module based on YOLOv3 for target detection in hazy and low-light situations. Li et al. [18] proposed a lightweight de-fogging network, and integrated it with the Faster R-CNN network to improve average detection accuracy. Jiang et al. [19] introduced a multi-scale progressive fusion network (MSPFN) for single image de-raining and it improved detection accuracy a lot compared to the original rain image. Stanley Zhang et al. [20] proposed ways to improve the YOLOv7 algorithm. They replaced the backbone network with MobileNetv3 to make it lighter. They used attention mechanisms to stop noise and interference. And they integrated bidirectional weighted feature pyramids to make feature aggregation better. Hnewa et al. [21] presented a cross-domain target detection method using multi-scale features and domain adaptive approaches. Liu et al. [22] proposed an IA–YOLO image adaptive target detection network. It includes a differentiable image processing module with adaptively learned hyperparameters. And it achieved good results. Oreski [23] proposed the YOLO*C algorithm. This algorithm introduced the Multi ConTeXt (MCTX) context module and improved the loss function. It improved the problems of vehicle and traffic sign detection under insufficient light and in complex environments. Özcan et al. [24] introduced the Gray Wolf Optimizer (GWO), Artificial Rabbit Optimizer (ARO), and Chimpanzee Leader Selection Optimization (CLEO) into the YOLO model. These algorithms significantly improved the detection accuracy of object detection in adverse weather conditions (AWCs). Ding et al. [25]. Designed the CF-YOLO model. They put forward a novel Cross-Fusion (CF) module, which can handle adverse detection problems such as the blurring, distortion and coverage of targets in snowy conditions.

Although previous methods have improved target detection in adverse weather conditions, most are limited to handling just one type of adverse weather. In complex traffic scenarios, issues like low detection accuracy, frequent missed detections, and false detections still persist. Additionally, when detecting targets of different scales and types, the model’s feature fusion ability is often insufficient. Considering the applicability of the YOLO series network to traffic detection systems, this paper adopts the YOLOv7 network [26] as the foundation to solve the target detection problem in adverse weather conditions. We propose a novel weather target detection algorithm, which is inspired by the receptive field enhancement and cross-scale fusion (RFCS) concepts. This proposed detection algorithm is referred to as RFCS-YOLO. The proposed algorithm aims to improve the feature extraction and fusion capabilities of the backbone network for varying targets (e.g., the targets with different shapes, sizes, and orientations), while optimizing the loss function. Experimental results validate the effectiveness of the proposed RFCS-YOLO algorithm. In particular, the contributions of this paper are summarized as follows:

1. An efficient feature extraction module (EFEM) is designed to enhance the capacity of the backbone network in terms of the multi-scale vehicle target feature extraction and expand the receptive field of the detection model.
2. A high cross-scale feature fusion (CSF) module is proposed to filter out invalid background information, and facilitate the interaction of feature information across different dimensions and scales. This enables the neck network to acquire more precise information regarding the position and texture of the vehicles and pedestrians. Additionally, the integration of the P2 feature layer, which contains richer small target information, into the feature fusion network aids in retaining more effective information.
3. The loss function is enhanced by combining the principles of Minimum Point Distance Intersection over Union (MPDIoU) [27] and Focaler-Intersection over Union (Focaler-IoU) [28]. The refined F-MPDIoU loss function addresses the leakage and false detection problems, thereby improving the overall detection performance.

## 2. Proposed RFCS-YOLO Algorithm

### 2.1. Design of RFCS-YOLO Network Architecture

**Figure 1:**
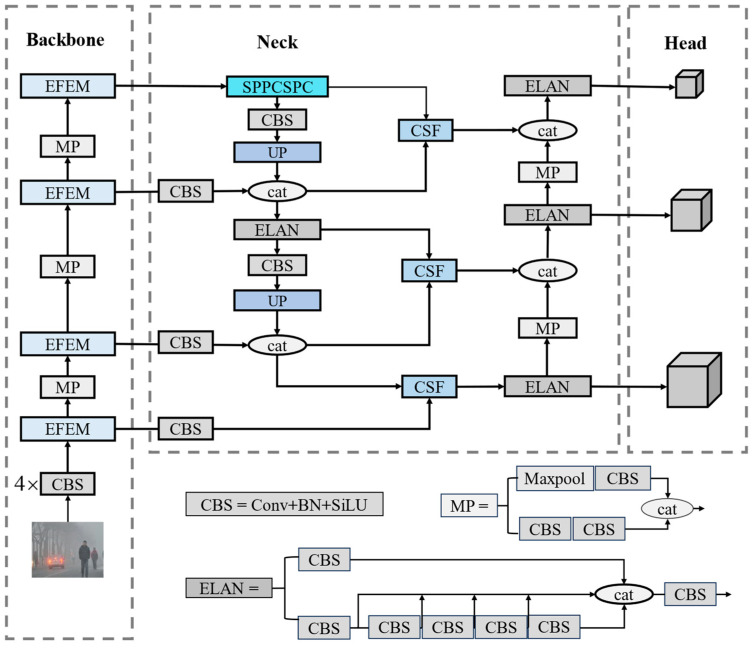
Overall network structure of RFCS-YOLO.

YOLOv7 is one of the most popular target detection algorithms in the YOLO series. It combines strategies like Efficient Layer Aggregation Networks (ELANs) [29], cascade model-based scaling, and model reparameterization. This is performed to strike a balance between detection efficiency and accuracy [30]. In this paper, we use YOLOv7 as a benchmark and propose an improved RFCS-YOLO network. The architecture of the proposed RFCS-YOLO network, depicted in Figure 1, includes an input module, a backbone network, a neck network, and a detection head. 

Firstly, the input undergoes preprocessing operations (e.g., data enhancement [31]) to resize the input image, meeting the requirements of the backbone feature extraction network. Then, the backbone network extracts key features from the input image, producing three feature maps of different scales. The backbone network primarily consists of the Efficient Feature Extraction Module (EFEM). The EFEM employs a gradient diversion strategy and partial convolutions (Pconv [32]) of diverse sizes to control the model parameters. This facilitates the extraction of features for targets of different scales [33]. Moreover, a deformable feature extraction branch utilizing deformable convolutions is designed. This design aims to compensate for the accuracy degradation caused by the reduction in parameters.

Next, the neck feature fusion network fuses three feature maps across multiple scales [34]. Within this network, we designed an innovative CSF module. This module helps reduce semantic degradation during feature transfer, while also filtering out interfering information [35]. As a result, the network focuses on effective information. Additionally, we introduced a new P2 feature layer to the fusion network. This layer provides richer information about small targets, enhancing the network’s ability to capture fine details.

Finally, the detection head carries out training and prediction. For optimization, prediction results and real labels are input into the loss function. We then apply non-maximum suppression to eliminate redundant detection boxes, ensuring accurate identification of vehicles and pedestrians.

### 2.2. EFEM

The vehicle and pedestrian images captured in adverse weather conditions reflects the following characteristics, including the blurred target edges, insufficient texture information, and significant scale changes. To deal with these issues, this paper proposes an Efficient Feature Extraction Module (EFEM). The EFEM is designed to improve the model’s adaptability to different scales and expand the receptive field for detecting targets [36]. This enhances the backbone network’s feature extraction capability for targets of various shapes and sizes.

**Figure 2:**
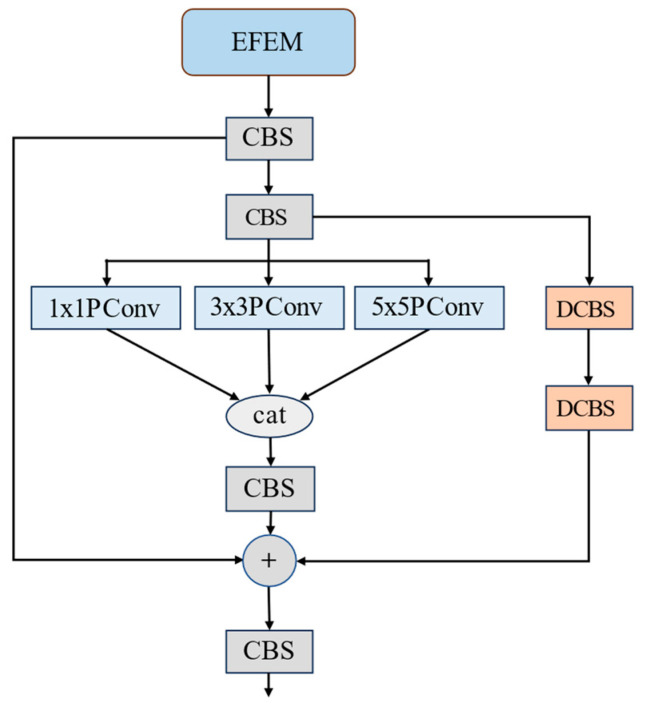
The structure of EFEM.

As depicted in Figure 2, the EFEM comprises three branches. The first branch includes a residual connection that boosts information transfer and improves the model’s generalization. The second branch uses partial convolution (Pconv) with kernel sizes of 1 × 1, 3 × 3, and 5 × 5. This allows for multi-scale feature extraction, reducing model redundancy and memory use. The outputs from these convolutions are then combined for channel-wise feature fusion [37]. The third branch consists of two deformable convolutional layers with offset learning capabilities. These layers dynamically adjust the size and shape of the sensing field based on the target. This allows for the precise detection of local details and adapts to targets of different shapes, sizes, and orientations. Finally, the output feature maps from the three branches are summed in the spatial dimension to yield the EFEM output.

**Figure 3:**
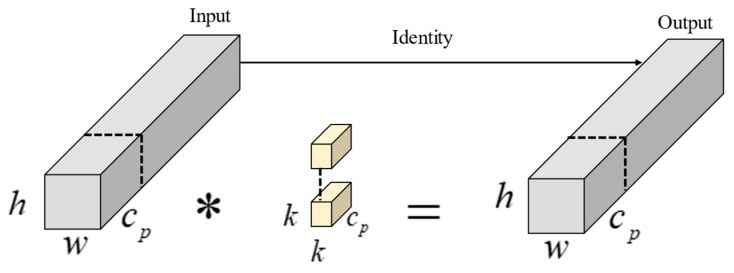
Pconv structure diagram. This asterisk represents convolution.

The partial convolution (Pconv) mechanism is illustrated in Figure 3. Feature maps generated from multiple convolutions of the input image often contain redundant features. To address this, Pconv selectively convolves only a portion of the input feature channels, leaving the rest unchanged. This approach reduces redundant computation and enables more efficient spatial feature extraction. The second branch of the EFEM consists of three Pconv convolutions with different kernel sizes. These convolutions generate feature maps of varying sizes. Pconv convolution only processes a portion of the input channels, which helps reduce redundant feature information. The three output feature maps of different sizes are then concatenated. Finally, the CBS layer adjusts the size of the output features.

For an input feature layer with a channel count, height, and width, denoted by c×h×w, the computational complexity of regular convolution is represented as $h\times w\times k^{2}\times c^{2}$, where *k* signifies the convolution kernel size. In contrast, the computational complexity of Pconv is represented as $h\times w\times k^{2}\times {c_{p}}^{2}$, where $c_{p}$ represents the subset of channels subjected to convolution. When considering $c_{p}=1/4$, the computational complexity of Pconv is merely 1/16th of that of conventional convolution.

Images captured in adverse weather conditions often have a non-uniform distribution due to weather disturbances. Additionally, vehicles and pedestrians in these images show significant variations in shape, scale, and pose. Traditional convolutions, which use fixed sizes and positions, struggle to adapt to these unknown variations and often have poor generalization. To address this, deformable convolution v2 (DCNv2 [38]) is applied to extend sampling across a wider range of features, improving the model’s adaptability to different scales. Therefore, we introduced a deformable convolution branch in the EFEM. This branch consists of a deformable convolution layer, a batch normalization layer, and a SiLU activation function. It is primarily responsible for extracting features of vehicles with various shapes and pedestrians in different postures. This addition helps the EFEM capture more accurate boundary information of the detection targets. As a result, the model can learn more comprehensive feature details.

**Figure 4:**
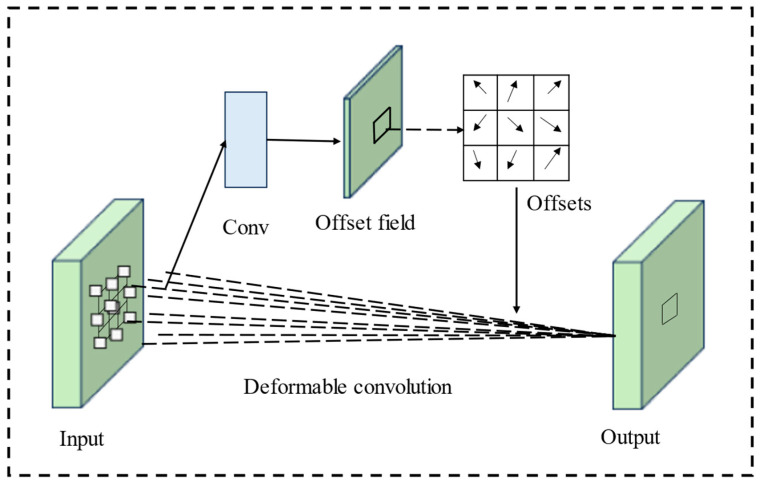
Deformable convolution feature extraction process.

As shown in Figure 4, in DCNv2, deformable convolution introduces an offset to each sampling point in the convolutional kernel, allowing the kernel to take on arbitrary shapes. During training, the model learns both the offset and an associated weight for each sampling point, which determines the influence of that point on feature extraction. The computation for deformable convolution is expressed as follows:

(1) $$y(P_{0})=\sum_{P_{n}\in R}\omega \left(P_{n}\right)\cdot x\left(P_{0}+P_{n}+\Delta P_{n}\right)\cdot \Delta m_{n}$$

where R is the regular grid of convolution kernel. Taking 3 × 3 convolution as an example, $R=\left\{\left(-1,1\right),\left(-1,0\right),\ldots ,\left(0,1\right),\left(1,1\right)\right\}$. $P_{0}$ is the pixel positions of the feature map. $P_{n}$ is the position in *R*, and $\Delta P_{n}$ is the offset ($\left\{\Delta P_{n}|n=1,\ldots ,N\right\}$), where N=|R|. *x* denotes the input feature map, and *y* denotes the output feature map. The weight of the sampling position is represented as ω, and $\Delta m_{n}$ is a one decimal number between [0, 1].

### 2.3. CSF Module

In the multi-scale feature maps generated by the Backbone Network, low-scale features have higher resolution, which aids in precise target localization. In contrast, high-scale features have lower resolution but are rich in semantic information. By combining these shallow and deep features, the model’s feature representation capability improves, leading to enhanced overall performance [39].

The YOLOv7 model uses the Path Aggregation Network (PAFPN) [40] structure, which adds an extra bottom-up pathway on top of the traditional Feature Pyramid Network (FPN) [41]. This addition allows information to flow both top-down and bottom-up, addressing the issue of limited information in low-dimensional features within the high-dimensional feature space. However, this approach simply adds multiple feature layers, mixing both useful and irrelevant information. Additionally, the repeated cross-scale feature transfer and multiple up-sampling steps can cause semantic information loss and degradation at different levels. To address this, we propose the Cross Scale Feature Fusion Module (CSF) to improve the PAFPN feature pyramid network.

**Figure 5:**
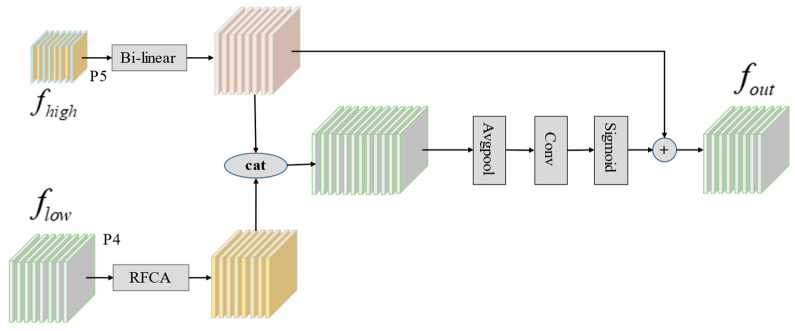
The structure of CSF module.

As shown in Figure 5, the CSF creates efficient connections between feature layers at different scales. This improves interaction between low-dimensional positional information and high-dimensional semantic information, enhancing feature fusion across dimensions. First, the CSF module uses the RFCA mechanism [42] to filter out noise and complex backgrounds in low-scale feature maps, while also expanding the receptive field. Then, to align the dimensions of high-level and low-scale features, bilinear interpolation is used to up- or down-sample high-level features. Finally, the filtered low-scale features are fused with high-scale features to boost the model’s feature representation capability. To enhance the feature pyramid network’s ability to capture accurate target location information, we add the P2 feature layer from the Backbone network. This addition provides the model with a more comprehensive set of feature information.

**Figure 6:**
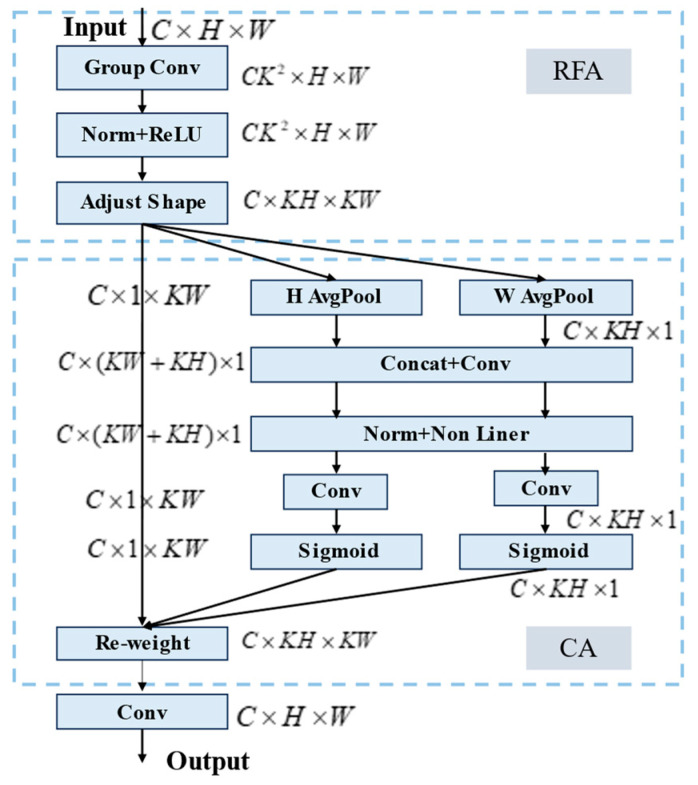
RFCA mechanism.

Receptive-Field Coordinate Attention (RFCA) is a module that enhances the lightweight Coordinate Attention (CA) [43] with a receptive field attention mechanism. This mechanism directs attention to the spatial features within the receptive field, addressing the issue of parameter sharing in convolutional kernels. The RFCA module highlights the importance of various features within the receptive field. It also prioritizes spatial features to capture long-distance information, similar to the self-attention mechanism. This approach addresses the limitation of the CA mechanism, which only focuses on spatial features. It does this while keeping computational costs low. Additionally, it effectively solves the problem of convolutional parameter sharing, enhancing the model’s focus on contextually significant information. The improved RFCA mechanism, as shown in Figure 6, first uses grouped convolution to extract spatial features from the receptive field to reduce feature overlap. Next, the adjusted spatial features are passed into the CA mechanism. This enhances the model’s ability to focus on key information while minimizing interference from complex contextual information, ultimately improving detection performance.

### 2.4. Improvement of Loss Function

The YOLOv7 algorithm employs the Complete Intersection over Union (*CIoU*) loss function [44] to compute the regression loss for prediction frames, as delineated by the formula presented below:

(2) $$IoU=\frac{B_{gt}\cup B_{prd}}{B_{gt}\cap B_{prd}}$$

(3) $$CIoU=IoU-\frac{\rho^{2}\left(B_{gt},B_{prd}\right)}{C^{2}}-\partial V$$

(4) $$V=\frac{4}{\pi_{2}}(\arctan\frac{w^{gt}}{h^{gt}}-\arctan\frac{w^{prd}}{h^{prd}}{)}^{2}$$

(5) ∂=V/(1−IoU+V)

In the CioU loss function used in the YOLOv7 algorithm, several key parameters are crucial for calculating regression losses for prediction frames. Specifically, $\rho^{2}\left(B_{gt},B_{prd}\right)$ represents the Euclidean distance between the prediction box and the centroid of the actual box. $C^{2}$ signifies the diagonal length of the smallest bounding rectangle. Additionally, ∂ denotes the equilibrium coefficient, and V serves as a metric for evaluating the aspect ratio disparity between the real and predicted boxes. The CioU loss function employs a relative value to characterize the aspect ratio relationship between the real and predicted boxes. Notably, the penalty term of the CioU function becomes ineffective when the aspect ratios of the real and predicted boxes are equal. This phenomenon leads to slow convergence of the model and yields unsatisfactory results.

To effectively utilize the geometric characteristics of bounding boxes in the loss function, the MPDIoU loss function was introduced. MPDIoU simplifies the comparison of predicted and actual bounding boxes. It does this by directly predicting the minimum distance between their upper-left and lower-right corners. This method works for both overlapping and non-overlapping bounding box regression tasks. It can improve the model’s convergence speed and detection accuracy. The formulation of the MPDIoU loss function is presented below:

(6) $$MPDIoU=IoU-\frac{\rho^{2}\left(A^{prd},A^{gt}\right)}{w^{2}+h^{2}}-\frac{\rho^{2}\left(B^{prd},B^{gt}\right)}{w^{2}+h^{2}}$$

In the formula mentioned above, $A^{gt}$, $B^{gt}$, $A^{prd}$, and $B^{prd}$ denote the coordinates of the upper-left and lower-right corners of both the true and predicted frames. $\rho^{2}\left(A^{prd},A^{gt}\right)$ and $\rho^{2}\left(B^{prd},B^{gt}\right)$ signify the Euclidean distance between these pairs of points.

However, there is a significant issue of sample imbalance during the detection process. To allocate gradients more reasonably and improve detection accuracy, we incorporated the idea of Focaler-IoU into the MPDIoU loss function. This led to the design of the F-MPDIoU loss function. 

F-MPDIoU includes factors such as overlapping and non-overlapping regions, distances between center points, and width and height deviations. Additionally, F-MPDIoU addresses the problem of sample imbalance during training. Samples are divided into easy and hard subsets based on detection difficulty. When easy samples dominate, more attention is given to hard samples, and vice versa. The problem of sample imbalance during training is efficiently handled using a linear interval mapping method, making gradient allocation more reasonable. The formula is as follows:

(7) $$L_{F-MPDIoU}=L_{MPDIoU}+IoU-IoU^{Focaler}$$

(8) $$L_{MPDIoU}=1-MPDIoU$$

(9) $${IoU}^{Focaler}=\left\{\begin{array}{ll} 0, & IoU<d\\ \frac{IoU-d}{u-d}, & d\leq IoU\leq u\\ 1, & IoU>u \end{array}\right.$$

where $\left[d,u\right]\in \left[0,1\right]$, by adjusting the values of d and u, $IoU^{Focaler}$ can be made to focus on different regression samples.

## 3. Experiment Results

### 3.1. Experiment Setup and Evaluation Metrics

The DAWN dataset [45] is adopted in the simulations. It comprises 1027 real traffic images captured under four types of adverse weather conditions: rain, snow, fog, and dust. These images are annotated using LabelMe 4.5.6for various traffic entities, including cars, buses, trucks, motorcycles, bicycles, and pedestrians. However, due to the limited number of motorcycles, bicycles, and buses in the original dataset, they only constitute 3.41% of the dataset. To ensure the reliability of the experiment, we manually annotated an additional 473 road traffic images. These images feature motorcycles, bicycles, and buses in rainy, snowy, foggy, and dusty conditions. The annotations were conducted using LabelMe. Subsequently, the dataset was augmented with data, resulting in a total of 1500 images. Finally, these images were further augmented, yielding a dataset comprising 5000 experimental images. These images were randomly divided into training, testing, and validation sets in a ratio of 7:2:1.

The experiments were conducted on a platform built on PyTorch 1.10.0 and CUDA 11.8, with the experimental operating system being Ubuntu 20.04. The GPU utilized was an NVIDIA GeForce RTX 3090, equipped with 24 GB of video memory. The core chip of this GPU is designed by NVIDIA, a technology company headquartered in Santa Clara, California, USA. Model training employed the SGD optimization strategy for a duration of 300 epochs, with the image size set to 640 × 640 and a Batch Size of 4. Additionally, a weight decay coefficient of 0.0005 and a learning rate momentum of 0.937 were applied.

In this experiment, Precision (P), Recall (R), Average Precision (AP), and Mean Average Precision (mAP) serve as the evaluation metrics to assess the performance of the proposed RFCS-YOLO algorithm. The formulas above metrics are, respectively, formulated as:

(10) $$P=\frac{TP}{TP+FP}$$

(11) $$R=\frac{TP}{TP+FN}$$

(12) $$AP=\int_{0}^{1}P\left(R\right)dR$$

(13) $$mAP=\frac{1}{N}\sum_{1}^{N}AP$$

where TP represents the true positive samples of categories correctly predicted as positive by the model; FP represents the false positive samples of categories incorrectly predicted as positive by the model; FN represents the false negative samples of categories incorrectly predicted as negative by the model; and N denotes the total number of categories.

### 3.2. Ablation Experiment

To validate the efficacy of the proposed enhancement approach, ablation experiments were conducted for each module using the expanded DWAN. Throughout these experiments, we ensured a standardized experimental environment and parameter configuration. Precision (P), Recall (R), Average Detection Precision (mAP@0.5), and the Number of Parameters (Params) were chosen as the evaluation metrics. The specific experimental outcomes are detailed in Table 1.

As observed from the table, experimental groups 1–7 underwent quantitative analysis employing different combinations of modules. Group 1 represents the original YOLOV7 experiment results and serves as the benchmark model. It has a precision (P) of 79.9%, a recall (R) of 78.8%, and an mAP@0.5 of 82.3%. The parameter count is 37.1 M. Groups 2 and 3 incorporate the EFEM and CSF modules into the model, respectively. This leads to a more noticeable improvement in the model’s precision rate; however, the recall rate decreases. In Group 2, the ELAN module in the backbone network is replaced with the designed EFEM. This change reduces the number of model parameters to 33.4 million due to the multiscale Pconv branching. Additionally, introducing the deformable branching results in a 1.1 percentage point increase in mAP@0.5. In Group 3, the neck of the original model is enhanced with our designed CSF module. This enhancement allows the model to acquire more comprehensive feature information through the cross-scale feature fusion module. As a result, there is a 1.3 percentage point increase in mAP@0.5. In Group 4, with the addition of the F-MPDIoU loss function, achieves the best result among all the experiments in terms of model recall. In Group 5, with the combined effect of the EFEM and the CSF module, mAP@0.5 improves by 2.4 percentage points. However, recall decreased by 1.9 percentage points. This indicates that the combination of the designed Efficient Feature Extraction Module and the Cross-Scale Feature Fusion Module can produce good detection results. However, it cannot effectively address the issue of missed detections. In Group 6, using the CSF module along with the F-MPDIoU loss function, leads to a more significant improvement in the model’s recall. This suggests that the improved loss function can effectively mitigate the issue of missed detections. Finally, in Group 7, all the improved methods are used together. As a result, precision, recall, and mAP@0.5 are enhanced by 8.3%, 1.4%, and 4.2%, respectively, compared to the original model. Additionally, there is a decrease in the parameter count relative to the original model.

To determine the optimal attention mechanism within the CSF module, this paper integrates the CSF module with three classic attention mechanisms: Channel attention mechanism Squeeze-and-Excitation module (SE) [46], coordinate attention (CA), and the Convolutional Block Attention Module (CBAM) [47], which combines channel and spatial information. Next, we conduct a comprehensive comparison with our proposed RFCA mechanism using the DWAN. The experimental results are summarized in Table 2.

It can be observed from Table 2 that, using the YOLOv7 model as the benchmark, when no attention mechanism is added to the CSF module we designed, the improvement in the model’s mAP@0.5 is not significant. However, after introducing attention mechanisms, the model generally shows an improvement in the accuracy (P) metric. At the same time, the recall (R) metric shows a downward trend. This indicates that the number of false detections of traffic targets has decreased, but the number of missed detections has slightly increased. The main reason for this is that attention mechanisms help the model focus more on key information. They reduce the chances of background noise being misidentified as correct targets. However, these mechanisms can also cause the model to overlook relatively subtle information, which may impact the accurate prediction of some targets. In terms of mAP@0.5 and the number of parameters, the RFCA mechanism outperforms the other attention mechanisms. It demonstrates the best detection accuracy with a reasonable increase in parameters.

### 3.3. Loss Function Comparison

**Figure 7:**
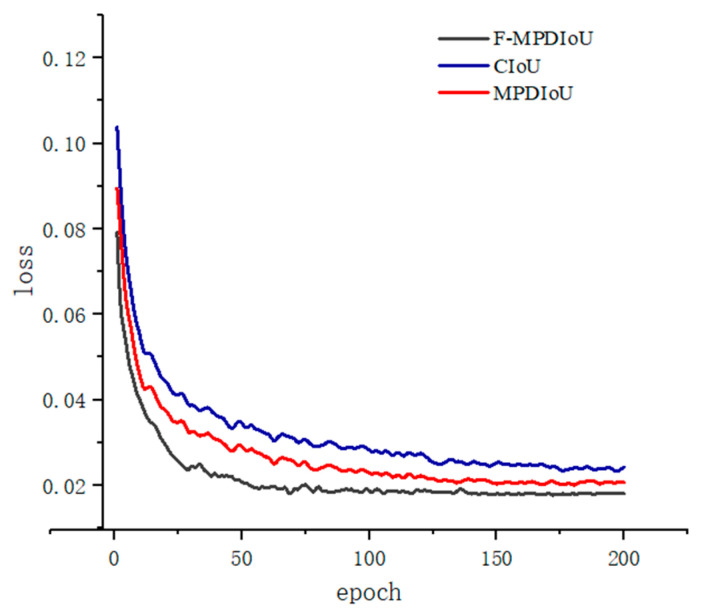
Loss function comparison chart.

To verify the effectiveness of the F-MPDIoU loss function proposed in this paper, we trained models using CioU, MPDIoU, and F-MPDIoU. This training was conducted for 200 epochs on the DWAN under the same experimental conditions. A comparison of the visualized loss function results is shown in Figure 7. The figure shows that the improved F-MPDIoU loss function yields the best results. MPDIoU performs the second best, while the original CioU loss function from YOLOv7 performs the worst. Notably, the loss values for both F-MPDIoU and MPDIoU dropped below 0.03 after 50 epochs. In contrast, the CioU loss function did not reach this level until much later. This indicates that F-MPDIoU and MPDIoU enhance the model’s convergence speed and accuracy compared to CioU.

After 200 epochs, the final loss values for CioU, MPDIoU, and the improved F-MPDIoU were 0.024, 0.021, and 0.018, respectively. Therefore, the improved F-MPDIoU was selected as the bounding box loss function for this study. The F-MPDIoU loss function effectively leverages the geometric properties of the bounding box. It also addresses the issue of uneven sample difficulty in adverse weather conditions through linear interval mapping. This approach results in higher training accuracy and faster convergence speed for the model.

### 3.4. Visual Analysis of Experimental Results

To illustrate the improvement effect more clearly, we compared the RFCS-YOLO algorithm with the original YOLOv7 algorithm. We trained and visualized the detection results on the expanded DWAN. We compared and analyzed the detection performance in two types of scenarios. The first scenario involved simple scenes with a small number of targets. The second scenario featured complex scenes with densely occluded targets. This analysis was conducted under four common adverse weather conditions: rain, snow, haze, and sand and dust.

**Figure 8:**
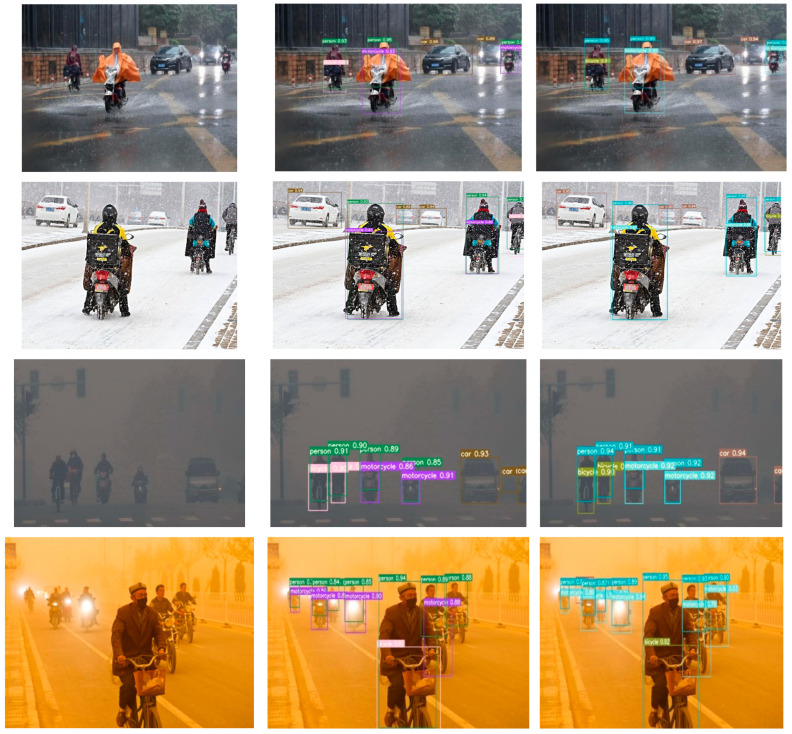
Comparison of detection results in simple scenarios.

**Figure 9:**
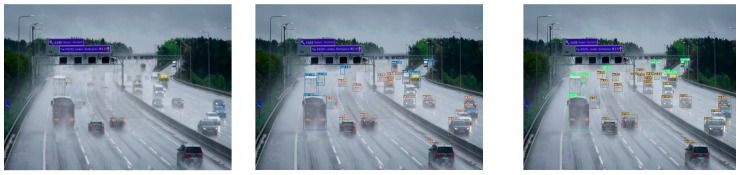

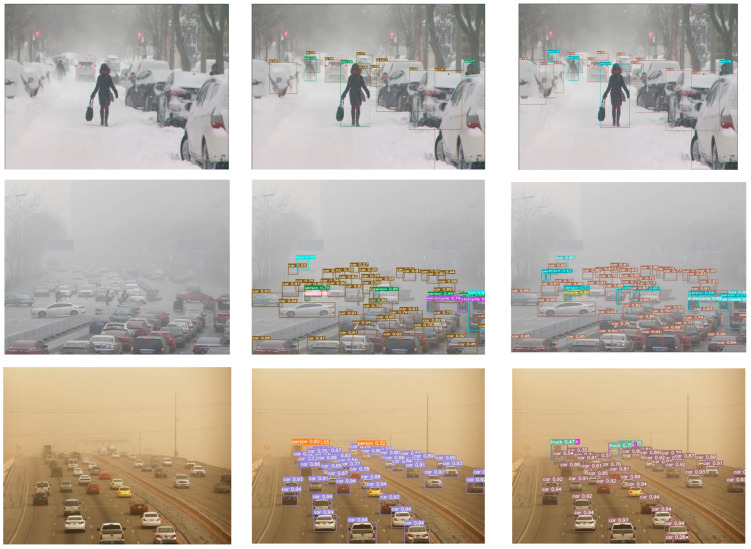
Comparison of Detection Results in Complex Scenarios with Densely Occluded Targets.

The comparison of detection results is presented in Figure 8 and Figure 9. In each figure, the first column shows the original images. The second column presents the detection outcomes from the original YOLOv7 model. The third column displays the detection results obtained with the RFCS-YOLO algorithm proposed in this paper. Figure 8 illustrates the detection performance in simple settings with a small number of targets. In contrast, Figure 9 demonstrates the detection outcomes in complex scenarios with densely occluded targets.

In the detection effect comparison chart presented in Figure 8, it is evident that in simple scenes with a limited number of detection targets, the original YOLOv7 algorithm yields satisfactory results. However, its detection accuracy falls short compared to our proposed RFCS-YOLO algorithm. This observation underscores the RFCS-YOLO algorithm’s ability to reduce interference from complex background details in adverse weather conditions. Consequently, it enhances the accuracy of vehicle and pedestrian detection.

It is clearly evident from Figure 9 that the YOLOv7 algorithm shows significant instances of misdetections and omissions in complex scenes with dense occlusions of vehicles and pedestrians. For instance, in rainy and snowy conditions, numerous small target vehicles at a distance and those obscured by precipitation are prone to misdetections. Similarly, in hazy weather, pedestrians and vehicles in densely shaded scenes can cause missed detections. Moreover, in sandy and dusty weather conditions, distant trucks are recognized as humans. In contrast, our proposed RFCS-YOLO algorithm effectively addresses issues of leakage and misdetection. It delivers better detection results even with targets of different shapes and scales. This improvement is evident in complex scenarios with dense occlusions.

**Figure 10:**
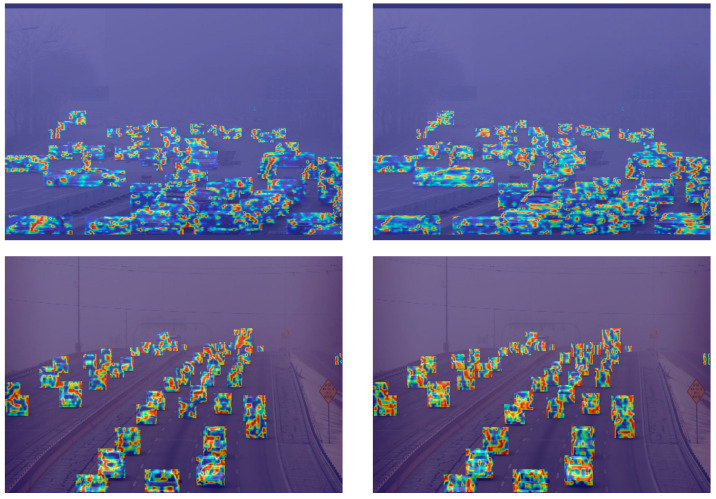
Heatmap comparison of detection results between YOLOv7 and RFCS-YOLO.

To visually observe the features learned by the trained network, we conducted a heatmap analysis on both the baseline model YOLOv7 and the RFCS-YOLO model. The results are shown in Figure 10. The first column displays the heatmaps from the baseline model, while the second column shows the heatmaps from the RFCS-YOLO model. From the figure, we can see that in dense scenes with occluded targets under adverse weather conditions, the baseline model exhibits insufficient feature learning. In contrast, RFCS-YOLO learns more detailed texture features of the targets and allocates more attention to them.

### 3.5. Experimental Results of Different Models Comparison

To further validate the effectiveness of the RFCS-YOLO algorithm proposed in this paper, we kept the experimental conditions and parameter settings consistent. We then compared our algorithm with other mainstream algorithms using the DWAN. The experimental results shown in Table 3 reveal that our algorithm achieves the highest mAP@0.5 of 86.5%. This represents a 4.2% improvement over the benchmark model, which is the YOLOv7 algorithm. Compared to the classical two-stage detection algorithm Faster R-CNN, our algorithm demonstrates significantly faster detection speed. Faster R-CNN is constrained by the sequential generation of candidate regions. It then follows up with classification, which slows down the overall process. Compared to the anchorless frame detection algorithm ConterNet [48], our algorithm performs better. Our algorithm can effectively handle overlapping targets of vehicles and pedestrians in the image. Compared to SSD, YOLOv5 [49], and YOLOv8 [50], our algorithm shows fewer cases of missed detections and misdetections. These three algorithms are mainstream one-stage target detection methods. Our algorithm is particularly effective at detecting multi-scale vehicles and pedestrians. Compared with the latest single-stage detection algorithms this year, namely YOLOv9, YOLOv10, and YOLOv11, the average detection accuracy of our model has been increased by 1.9%, 2.5%, and 1.1%, respectively. Although the frames per second (fps) is slightly lower than that of YOLOv10 and YOLOv11, the average detection accuracy of our model is higher than theirs. This is because these latest algorithms did not take into account the situation in complex weather environments such as haze, rain, and snow at the beginning of their design. In such environments, the collected images are blurry and there are occlusion problems for the detection targets. Regarding Real-Time Detection Transformer (RT-DETR) [51], its performance is hindered by interference under adverse weather conditions, making its detection less effective compared to our algorithm. Compared with the DETR algorithm [52], due to the complexity of the transformer architecture itself, its computational complexity will result in a slower detection speed, making it unsuitable for real-time target detection.

### 3.6. Adaptability and Generalization Comparison Experiment

To verify the adaptability and generalization ability of the RFCS-YOLO algorithm, we conducted a comparison experiment using the SODA10M [53] autonomous driving dataset. This dataset was jointly released by Huawei Noah’s Ark Laboratory and Sun Yat-sen University. It covers diverse scenes, including urban areas, highways, and urban-rural roads. The dataset also includes images taken under various weather conditions, such as sunny, cloudy, rainy, and snowy days. In terms of time span, the dataset features road scene photos taken at different times of day, including daytime, night, early morning, and dusk. The dataset primarily consists of six human–vehicle scene categories: Pedestrian, Cyclist, Car, Truck, Tram, and Tricycle. To ensure the accuracy and reliability of the experimental results, we maintained consistency in experimental conditions and parameter settings to minimize the potential impact of unrelated variables. In addition, to comprehensively and objectively evaluate the performance of the RFCS-YOLO model, we selected accuracy, recall, mAP@0.5, and FPS as the core evaluation metrics. To further investigate the learning ability of the RFCS-YOLO model, we chose not to use pre-trained weights from other datasets for training. Although pre-trained weights can accelerate model convergence in some cases, they may introduce specific biases and prior knowledge from other datasets, potentially interfering with the assessment of the model’s native learning capability on the target dataset. Therefore, we opted to use the labeled data provided by the SODA10M dataset and adopted a supervised training approach to train the model from scratch. We divided the dataset into training, validation, and test sets, with a ratio of 6:2:2. The training set contains 12,000 images, while both the validation and test sets include 4,000 images each. We set the batch size to 8 and the number of training epochs to 100. The training results are shown in Table 4.

As shown in Table 3, the mAP@0.5 of the RFCS-YOLO algorithm proposed in this paper reaches 56.7%. This represents a significant improvement compared to other algorithms. In terms of real-time detection performance, the frame rate of our algorithm reaches 58 frames per second, which is notably better than most other algorithms, except for YOLOv5. In summary, the RFCS-YOLO algorithm demonstrated in this paper exhibits strong adaptability and generalization capabilities in the field of traffic target detection.

## 4. Conclusions

To ensure robust target detection in adverse weather conditions like rain, snow, haze, sand, and dust, we propose the RFCS-YOLO algorithm. This algorithm also aims to improve detection accuracy for densely occluded and multi-scale targets. Firstly, we designed an efficient enhanced feature extraction module. This module strengthens the backbone network’s ability to model multi-scale targets and broadens its receptive field. Secondly, we use a cross-scale feature fusion module. This module filters out interfering information in the feature map and thoroughly integrates semantic information across different dimensions. Lastly, the F-MPDIoU loss function is introduced to prioritize training samples with differing difficulty levels, thereby enhancing model convergence speed. Experimental evaluations on the expanded DWAN and the autonomous driving dataset SODA10M demonstrate that our algorithm achieves exceptional detection accuracy and strong generalization ability. It outperforms other mainstream algorithms, especially in complex weather conditions. The algorithm effectively reduces vehicle and pedestrian omissions and misdetections, while capturing relevant feature information for targets of various scales. In future research, we plan to further expand the dataset by incorporating autonomous driving scene images from various weather conditions and environments. This will help improve the model’s generalization ability. Additionally, we aim to explore techniques such as knowledge distillation, network pruning, and quantization compression. These methods are intended to reduce model complexity and facilitate easier deployment on small embedded devices.

## Figures and Tables

**Table 1:** Ablation experimental results.

Groups	EFEM	CSF	F-MPDIoU	P (%)	R (%)	mAP@0.5 (%)	Params (m)
1				79.9	78.8	82.3	37.1
2	✓			85.6	77.6	83.4	33.4
3		✓		83.4	76.4	83.6	39.6
4			✓	85.2	80.8	83.1	37.1
5	✓	✓		87.2	76.9	84.7	35.7
6		✓	✓	86.8	80.6	84.5	39.5
7	✓	✓	✓	88.2	80.2	86.5	36.2

**Table 2:** Results of the ablation experiment on attention mechanisms.

Groups	Model	P (%)	R (%)	mAP@0.5 (%)	Params (m)
1	YOLOv7	79.9	78.8	82.3	37.1
2	CSF	80.4	78.5	82.7	37.5
3	CSF-SE	80.8	77.3	82.6	38.4
4	CSF-CBAM	81.6	78.4	83.0	41.9
5	CSF-CA	82.5	76.9	83.1	39.3
6	CSF-RFCA	83.4	76.4	83.6	39.6

**Table 3:** Experimental results of different models comparison.

Model	P (%)	R (%)	mAP@0.5 (%)	FPS (f/s)
Faster R-CNN	70.4	65.4	69.6	12
SSD	64.3	55.6	63.2	38
ConterNet	75.5	69.1	73.7	54
YOLOv5	81.0	73.4	79.7	73
YOLOv7	79.9	78.8	82.3	65
YOLOv8	85.3	76.5	80.3	70
YOLOv9	86.6	77.8	84.6	60
YOLOv10	84.1	79.2	84.1	71
YOLOv11	88.8	78.6	85.4	75
RT-DETR	83.2	76.7	81.7	66
DETR	84.1	75.8	83.2	35
RFCS-YOLO	88.2	80.2	86.5	68

**Table 4:** Comparative Experiment of generalization.

Model	P (%)	R (%)	mAP@0.5 (%)	FPS (f/s)
Faster R-CNN	46.7	42.4	41.4	11
YOLOv5	45.9	54.5	49.5	61
YOLOv7	53.1	46.2	53.1	52
YOLOv8	55.3	43.5	51.2	57
YOLOv11	57.1	50.6	54.6	55
DETR	51.5	55.2	52.9	23
RT-DETR	52.4	57.3	53.2	42
RFCS-YOLO	59.2	52.5	56.7	58

## Data Availability

The data presented in this study are available on request from the corresponding author after obtaining permission of an authorized person.
